# Editorial: New approaches to evaluation and management of dysphagia in neurological disease

**DOI:** 10.3389/fneur.2024.1467206

**Published:** 2024-08-14

**Authors:** Masahiro Nakamori, Omar Ortega Fernández, Jun Kayashita, Mineka Yoshikawa

**Affiliations:** ^1^Department of Clinical Neuroscience and Therapeutics, Hiroshima University Graduate School of Biomedical and Health Sciences, Hiroshima, Japan; ^2^Gastrointestinal Physiology Laboratory, Hospital de Mataró, Universitat Autònoma de Barcelona, Mataró, Spain; ^3^Centro de Investigación Biomédica en Red de Enfermedades Hepáticas y Digestivas (CIBERehd), Barcelona, Spain; ^4^Department of Health Sciences, Faculty of Human Culture and Sciences, Prefectural University of Hiroshima, Hiroshima, Japan; ^5^Department of Advanced Prosthodontics, Hiroshima University Graduate School of Biomedical and Health Sciences, Hiroshima, Japan

**Keywords:** dysphagia, rehabilitation, novel evaluation, novel instrument, neurophysiology, neuroimaging

Neurological diseases frequently cause dysphagia and consequent aspiration pneumonia, significantly impacting patient mortality, morbidity, and healthcare costs. It is therefore essential to implement better strategies for evaluating, addressing, and rehabilitating dysphagia. Several protocols and tools have been developed for dysphagia assessment, with videofluoroscopy and videoendoscopy being considered the gold standards (1). Moreover, recent advancements offer promising non-invasive and simple procedures and instruments for dysphagia assessment (2, 3). Effective rehabilitation protocols have also been established for clinical practice (4). Novel techniques, such as transcutaneous electrical stimulation of the neck, are being introduced and are expected to improve neuromuscular function (5, 6).

The utility of novel dysphagia assessment tools has recently been reported (7, 8). Nakamori, Ishikawa et al. investigated the use of electronic stethoscopes and artificial intelligence (AI) analysis for swallowing sound evaluation in patients with amyotrophic lateral sclerosis, suggesting potential applications in telemedicine and home care. Among these advancements, tongue pressure has gained significant attention due to its practical value. Fukuoka et al.'s minireview summarized the detailed relationship and limitations of tongue pressure and dysphagia in patients with Parkinson's disease (PD). Physiological functional imaging also offers new possibilities for evaluating dysphagia. Gu et al. focused on cortical compensation mechanisms in dysphagia following medullary infarction. Using blood-oxygen-level dependent functional magnetic resonance imaging, they observed increased activation in specific brain regions, including the bilateral precentral gyrus, postcentral gyrus, insula, thalamus, and supplementary motor areas, after rehabilitation. Notably, their study showed that effortful swallowing activated more brain regions than regular saliva swallowing, suggesting its effectiveness as an approach for rehabilitation. As previously mentioned, gold-standard evaluations are crucial, especially with a serious condition such as dysphagia. Cui et al. emphasized the importance of videofluoroscopic swallowing studies (VFSS) in dysphagia assessment, demonstrating significant differences in swallowing parameters between patients with PD and those with post-cerebral hemorrhage or infarction. They assert that standardizing quantitative VFSS parameters can improve the accuracy and consistency of dysphagia evaluation. Beyond evaluating for dysphagia, determining patients at risk of dysphagia is equally important in clinical practice. Karunaratne et al. reported that older individuals and patients with neurological disorders are more susceptible to developing or experiencing worsened complications, underscoring the need for proactive identification. Furthermore, a retrospective study by Choi et al. identified clinical predictors of dysphagia in patients with traumatic and non-traumatic cervical spinal cord injuries, focusing on forced vital capacity and tracheostomy as key predictors.

Several novel interventions on rehabilitation have emerged. Nakamori, Toko et al. investigated the effects of cervical percutaneous interferential current stimulation on dysphagia in patients with PD, reporting a significant improvement in oral cavity residue. Another study by Yeo et al. reported that therapeutic singing exercises may help maintain swallowing function and potentially improve swallowing-related quality of life in patients with advanced PD. The utility of acupuncture for dysphagia treatment has also been widely reported. Wu Y. et al. explored the combined use of postural control and electroacupuncture compared to conventional rehabilitation in 138 patients with post-stroke dysphagia. This integrative approach significantly improved swallowing function and reduced aspiration-related complications in the observation group than in the control group. This is further evidenced by the results of their standardized swallowing assessment and water swallowing test. Similarly, a systematic meta-analysis by Li et al. demonstrated the effectiveness and safety of acupuncture in treating aspiration among 1,284 patients with post-stroke dysphagia. Their findings revealed that acupuncture, used alone or combined with other therapies, significantly improved the Penetration Aspiration Scale scores, VFSS results, and hyoid bone displacement in these patients. Additionally, acupuncture showed a higher overall efficacy rate and fewer adverse events compared to conventional rehabilitation. In another study by Wu M. et al., data mining techniques were employed to analyze optimal acupuncture parameters for post-stroke dysphagia in 39 studies, which included needle size, retention time, treatment frequency, and core acupoints.

Comprehensive reviews on dysphagia and its guidelines have also been conducted. Bibliometric studies by Ye et al. and Guo et al. highlighted the evolving research trends in the study of dysphagia and stroke, such as aspiration, gastroesophageal reflux disease, neuroplasticity, and non-invasive brain stimulation. Likewise, He et al. utilized bibliometrics to identify research hotspots and cutting-edge trends in the field of post-stroke dysphagia rehabilitation. In their study, the keywords “validity” and “non-invasive brain stimulation” emerged as the most significant words in post-stroke dysphagia rehabilitation research. Regarding guideline studies, Gao et al. critically appraised clinical practice guidelines for managing dysphagia after acute stroke, revealing gaps between evidence and practice, particularly in applicability and stakeholder involvement.

The evaluation and management of dysphagia in neurological diseases have progressed significantly due to innovative therapeutic approaches, improved diagnostic techniques, and comprehensive research. Integrating these new insights into clinical practice can enhance patient outcomes, reduce complications, and improve quality of life. Continued research and collaboration among healthcare professionals are essential to further refine these strategies and develop effective personalized treatment plans for patients with dysphagia.
